# Non‐Pharmacological Interventions for the Treatment of Lumbopelvic Pain in Pregnant Women: A Systematic Review Protocol Using the TIDieR Checklist

**DOI:** 10.1002/msc.70250

**Published:** 2026-07-28

**Authors:** Marília Caroline Ventura Macedo Leite, Mateus Dantas Azevedo de Lima, Rafaela Jéssica Silveira de Souza, Vanessa Patrícia Soares de Souza, Elizabel de Souza Ramalho Viana

**Affiliations:** ^1^ Department of Physiotherapy Postgraduate Program in Physiotherapy Federal University of Rio Grande do Norte (UFRN) Natal Brazil; ^2^ Department of Physiotherapy Postgraduate Program in Physiotherapy Faculty of Health Sciences of Trairi Federal University of Rio Grande do Norte (UFRN) Santa Cruz Brazil

**Keywords:** lumbopelvic pain, pregnancy, therapeutics

## Abstract

**Background:**

Lumbopelvic pain (LPP) is one of the most common complaints during pregnancy, with a reported prevalence of 63%. During pregnancy, treatment should preferably be non‐invasive. However, scientific evidence on non‐pharmacological interventions for pregnant women with LPP is still limited.

**Objective:**

To synthesise the available evidence on the effectiveness of interventions for LPP in pregnant women and to assess the quality and completeness of intervention reporting using the TIDieR checklist.

**Methods:**

Randomised controlled trials evaluating non‐pharmacological interventions in pregnant women of any gestational age diagnosed with lumbopelvic pain, including low back pain, pelvic pain, or combined pain, will be included. Review studies, observational studies, editorials, letters, commentaries, preprints, dissertations, and theses will be excluded. This systematic review protocol follows PRISMA‐P recommendations and is registered in PROSPERO (CRD420251061024). Searches will be conducted in PubMed, PEDro, Cochrane Library, Scopus, Web of Science, Embase, and LILACS using terms related to pregnancy, lumbopelvic pain, and non‐pharmacological interventions. Four independent reviewers will select studies and extract data using the TIDieR checklist to assess the completeness of intervention reporting. The certainty of the evidence will be assessed using the GRADE system, and the risk of bias will be assessed using the RoB 2 tool. Disagreements will be resolved by a fifth reviewer. The results will be synthesised, and a meta‐analysis will be performed if feasible.

**Conclusion:**

The results can enhance scientific knowledge, support evidence‐based clinical decision‐making, and contribute to improving care protocols in women's health and obstetrics.

## 1 Introduction

Lumbopelvic pain (LPP) is a highly prevalent condition among pregnant women, affecting approximately 63% of this population (Shanshan et al. 2024; Diez‐Buil et al. 2024). Among them, 11%–25% classify LPP as severe, representing one of the most common complaints reported during pregnancy (Haakstad et al. 2023). This condition encompasses lower back pain, pelvic girdle pain, or a combination of both (Sushil et al. 2023).

LPP can manifest from the region of the 12th rib to the gluteal fold or around the pubic symphysis (Yildirim et al. 2022). Specifically, lower back pain is located between the 12th rib and the gluteal fold, while pelvic girdle pain occurs in the region between the posterior iliac crest and the gluteal fold (Vleeming et al. 2008). In this context, pelvic pain is considered a specific form of low back pain, and may occur in isolation or in association with lower back pain. Additionally, this pain may radiate to the posterior thigh and, in some cases, manifest concomitantly or separately from pubic symphysis pain (H. Wang et al. 2021).

LPP is multifactorial and arises as a result of the dynamic interaction between physiological, psychological, and social factors (Gatchel et al. 2007; Beales et al. 2020; Haakstad et al. 2023). According to a study by Koca and Özer (2024), pregnancy‐related lumbopelvic pain occurs due to mechanical, systemic, and hormonal reasons. Biomechanical changes resulting from uterine growth can lead to anterior displacement of the centre of gravity, compensatory increase in lumbar lordosis, joint overload, rotation, and pelvic instability, contributing to greater mechanical stress on the lumbar spine (Baracho 2018; Kizito et al. 2023). However, not all pregnant women with LPP present with hyperlordosis or significant postural changes, suggesting the involvement of other pathophysiological mechanisms.

Hormonal factors are also widely investigated. Relaxin has been associated with increased ligamentous laxity and joint mobility in the pelvis and spine; oestrogen may influence joint flexibility and calcium homoeostasis in the musculoskeletal system; and progesterone is related to reduced smooth muscle tone (Baracho 2018; Daneau et al. 2021, 2025). However, the literature still does not demonstrate a consensus regarding the direct association between hormone levels and the occurrence or intensity of pelvic floor dysfunction (Daneau et al. 2021).

Therefore, increased joint mobility due to the effect of the relaxin hormone on collagen, weight gain leading to fatigue, the weight of the growing foetus, and overload may be related to the onset of LPP during pregnancy (Kizito et al. 2023). However, it is important to recognise all the biopsychosocial factors that can trigger LPP, which negatively affects the quality of life of pregnant women (Kizito et al. 2023).

Multiparous women with a history of LPP, whose gestational weight gain exceeds the US Institute of Medicine (IOM) guidelines, with a pre‐pregnancy body mass index (BMI) > 25, who smoke, use oral contraceptives, work strenuously, are of advanced age, experience pain during menstruation, are HIV positive, have no income, and reside in South Asia or the Middle East are at higher risk of developing this condition during pregnancy (Haakstad et al. 2023; Kizito et al. 2023; Robinson et al. 2024). On the other hand, regular maternal physical exercise, more than twice a week, is associated with a lower prevalence and intensity of pregnancy‐related LPP (Davenport et al. 2019; Haakstad et al. 2023).

The management of LPP during pregnancy should initially involve non‐pharmacological methods, such as relaxation techniques, deep breathing, physiotherapy, acupuncture, hot and cold compresses, and transcutaneous electrical nerve stimulation (Gutke et al. 2015; medicines in Pregnancy 2026). Regarding medications, the American College of Obstetricians and Gynecologists (2025) states that paracetamol is the analgesic of choice during pregnancy; its use should be judicious, considering the lowest effective dose for the shortest necessary period, due to potential risks as well as benefits.

In this context, the importance of multimodal approaches that integrate different safe and effective therapeutic modalities for pregnant women has been highlighted (George et al. 2013; S. Wang et al. 2025; Vesting et al. 2025). Prenatal exercise programs such as yoga, aerobic exercise, overall or group muscle strengthening, and the combination of aerobic and resistance training have proven particularly effective in reducing the intensity of LPP and are considered both preventive and therapeutic strategies (Ozdemir et al. 2015; Davenport et al. 2019).

A systematic review of physiotherapy interventions for pregnant women with LPP reported very limited evidence on the effectiveness of interventions such as water exercises, progressive muscle relaxation, specific pelvic tilt exercises, osteopathic manual therapy, craniosacral therapy, electrotherapy, and yoga, reinforcing the need for well‐described, replicable, and scientifically grounded interventions (Gutke et al. 2015). Other evidence indicated that stabilisation exercises involving the activation of abdominal, paravertebral, pelvic floor, and diaphragm muscles are more effective in relieving lumbopelvic pain, improving disability, and increasing the quality of life in pregnant women compared with standard treatment alone (Mamipour et al. 2023).

While the systematic review by Davenport et al. (2019) highlighted the potential benefits of non‐pharmacological interventions for pregnancy‐related LPP, it also emphasised important methodological limitations in the included studies. These limitations included small sample sizes, high dropout rates, low adherence to exercise‐based interventions, inadequate reporting, and a lack of information about the interventions. These problems weaken the certainty of the evidence and limit the interpretation of the true effectiveness of the interventions.

In addition to the methodological concerns of previous studies, insufficient reporting of intervention components further compromises reproducibility and clinical applicability. When essential details, such as dosage, frequency, progression, and adherence strategies, are poorly described, it becomes difficult for clinicians and researchers to replicate or implement the interventions in real‐world settings. Therefore, in addition to updating the evidence base, there remains a need to assess how interventions are reported in the literature to promote transparency, reproducibility, and transposition to clinical practice (Hoffmann et al. 2014).

The Template for Intervention Description and Replication (TIDieR) is a 12‐item checklist developed to encourage researchers to report interventions with sufficient detail to enable replication, thereby improving the quality of intervention reporting in clinical trials and promoting greater transparency and reproducibility. The TIDieR checklist was developed following the methodological framework for reporting guideline development recommended by the EQUATOR Network and should be used in conjunction with the CONSORT statement when reporting randomised clinical trials or with the SPIRIT statement when reporting clinical trial protocols (Hoffmann et al. 2014).

The low quality and incomplete description of interventions in studies with pregnant women with LPP limit their replication and reliable implementation in clinical practice (Davenport et al. 2019). To date, no systematic review has assessed the completeness of the description of physiotherapy interventions for pregnancy‐related LPP using the TIDieR checklist, which represents a relevant methodological gap in the literature. Therefore, this systematic review aims to synthesise the available evidence on the effectiveness of physiotherapy interventions for LPP in pregnant women and to assess the quality and completeness of the description of the interventions. Thus, it will contribute to the advancement of scientific research and to safe physiotherapy clinical practice in obstetrics.

## 2 Methods

### 2.1 Study Design and Registration

The present protocol follows the guidelines established by the Preferred Reporting Items for Systematic Review and Meta‐Analysis Protocols (PRISMA‐P) (Shamseer et al. 2015) (Supporting Information S1: Appendix S1). It has been registered in the International Prospective Register of Systematic Reviews (PROSPERO) under the title ‘*Physiotherapy Interventions for Treating Lumbopelvic Pain in Pregnant Women: A Systematic Review Using the TIDieR Checklist*’, registration number CRD420251061024.

### 2.2 Review Question

What are the characteristics and effectiveness of non‐pharmacological interventions for the treatment of lumbopelvic pain in pregnant women?

The research question was developed using the PICO strategy (Table 1).

**TABLE 1:** PICO acronym.

Meaning of the acronym:	Description
*p* (population)	Pregnant women
*I* (intervention)	Non‐pharmacological interventions
*C* (comparison)	Usual care, advice
*O* (outcome)	Lumbopelvic pain

### 2.3 Eligibility Criteria

#### 2.3.1 Inclusion Criteria

Studies will include pregnant women of any age and gestational trimester diagnosed with pregnancy‐related LPP, including low back pain, pelvic girdle pain, or combined pain. Eligible studies will consist of randomised clinical trials that evaluated the effectiveness of non‐pharmacological interventions for the treatment of LPP in pregnant women, without restriction of language or publication date.

#### 2.3.2 Exclusion Criteria

Studies involving pregnant women with pre‐pregnancy lower back, pelvic or LPP, herniated discs or vertebral disc degeneration prior to pregnancy, and neurological or cognitive disorders will be excluded.

#### 2.3.3 Exposure

Non‐pharmacological interventions such as exercise therapy, manual therapy, electrothermophototherapy, health education, motor control, stabilisation, Pilates, yoga, strengthening, resistance training, core stability, pelvic stabilisation and neuromuscular exercise are aimed at treating LPP during pregnancy.

### 2.4 Primary Outcomes

Intensity of LPP. Through the use of scales or questionnaires before and after interventions, for example, the Numeric Pain Rating Scale (NRS), Visual Analogue Scale (VAS), Pelvic Girdle Questionnaire (PGQ), Oswestry Disability Index (ODI).

### 2.5 Secondary Outcomes

Disability (PGQ, WHO Disability Assessment Schedule 2.0, ODI), quality of life, adverse events, absenteeism from work.

### 2.6 Search Strategy

The search strategy will include terms related to ‘pregnancy’, ‘lumbopelvic pain’, and ‘therapeutics’. The detailed search strategy for each database is provided in Supporting Information S2: Appendix S2. The principal investigator (MCVML) will apply the search strategy across all databases.

### 2.7 Information Sources

To achieve the proposed objective, searches will be conducted in the following databases: PubMed, PEDro, the Cochrane Library, Scopus, Web of Science, Embase, and LILACS.

### 2.8 Study Selection

The records retrieved from each database will be imported by the principal investigator into the Rayyan platform (Doha, Qatar) and used for the data management and screening process (Ouzzani et al. 2016). Initially, duplicate records will be removed. Subsequently, titles and abstracts will be screened, and the eligibility criteria will be applied to identify studies relevant to the review. Articles that meet the inclusion criteria will then undergo full‐text assessment.

This process will be conducted independently by four reviewers (MCVML, MDAL, RJSS, VPSS). In cases of disagreement regarding study inclusion or exclusion, a fifth reviewer (ESRV) will resolve discrepancies. When relevant data are missing or unclear, the corresponding author of the study will be contacted. All excluded studies, along with the reasons for exclusion, will be reported in the final review.

### 2.9 Data Extraction

After reviewing the full text and confirming the inclusion of the study, data extraction will be performed for each included article. This process will be conducted independently by the assigned reviewers using an Excel spreadsheet developed by the lead author (MCVML).

The data extracted from each study will include: first author, year of publication, country, study design, sample size, social class, ethnicity, mean age of participants, gestational age, presence and form of diagnosis of LPP, intervention and control groups, methodological procedures, and main outcomes. The characteristics of each intervention will be structured according to the TIDieR checklist in its original version (Hoffmann et al. 2014). The description of the intervention, theory or objectives of the essential elements of the intervention, materials used, procedures applied, professional who applied it, mode and location where it took place, number of sessions, schedule, duration, intensity or dose, and whether there were adaptations or modifications from what was planned to what was executed will be considered (Hoffmann et al. 2014).

### 2.10 Assessment of the Certainty of the Evidence

The certainty of the body of evidence and the strength of the recommendation will be assessed independently by four reviewers (MCVML, MDAL, RJSS, VPSS) using the GRADE assessment tools and recommendations (Guyatt et al. 2011). A fifth reviewer (ESRV) will be consulted in case of disagreement.

### 2.11 Risk of Bias

During the assessment of the risk of bias of each article, the four reviewers (MCVML, MDAL, RJSS, VPSS) will independently use version 2 of the Cochrane Risk of Bias tool for randomised trials (RoB 2), as described by Sterne et al. (2019), in accordance with the methodological recommendations of the Cochrane Handbook (Higgins et al. 2024). Any disagreements will be resolved by the fifth reviewer (ESRV).

### 2.12 Data Synthesis and Integration

All extracted results are summarised in tables and figures. Frequency tables indicate the proportion of studies that reported each item of the TIDieR. A qualitative synthesis of the intervention reporting pattern will be performed, identifying which components are most frequently omitted or described incompletely, as well as possible trends related to the type of intervention or year of publication. A PRISMA flowchart will illustrate the entire study selection process (Page et al. 2021). If a meta‐analysis is feasible, Jamovi software (Version 2.3.28; The Jamovi Project 2024) (https://www.jamovi.org/) will be used.

For continuous outcomes, mean differences (MD) will be used when the included studies present their results based on the same measurement scale. When different instruments are applied to analyse the same outcome, standardised mean differences (SMD) will be used. When more than one instrument is used to assess the same outcome in a study, a predefined hierarchy will be adopted. For pain, priority will be given to the VAS, followed by the NRS, and subsequently to other validated pain measures. For disability, priority will be given to the PGQ, followed by the ODI and other validated disability instruments.

In studies with multiple intervention arms, similar intervention groups will be aggregated when appropriate. For studies with multiple assessment points, the assessment at the time point closest to the end of the intervention will be considered. When both post‐intervention values and changes from baseline are reported, post‐intervention values will be preferred. Change scores will only be extracted when post‐intervention values are not available. Further analyses may be developed according to the follow‐up period of the studies, considering short (up to 12 weeks after the end of the intervention), médium (more than 12 weeks to 6 months), and long term (more than 6 months).

Clinical trials will be grouped according to the type of comparator used. Studies evaluating non‐pharmacological interventions compared to usual care, placebo, waiting list, or no intervention will be analysed separately from those using active comparators. Trials involving comparisons between two or more active interventions (e.g., exercise vs. stabilisation, exercise vs. yoga, or different manual therapy approaches) will be synthesised separately from studies with non‐active comparators when there is sufficient clinical and methodological homogeneity.

Multimodal interventions will be considered a distinct category of non‐pharmacological interventions. Their composition will be described based on information extracted from the TIDieR checklist, and grouping in meta‐analyses will only occur when the components and therapeutic objectives are sufficiently similar across studies.

Statistical heterogeneity will be assessed using the I^2^ statistic with a 95% confidence interval. The thresholds for interpreting heterogeneity will be defined as follows: 0%–40% unimportant heterogeneity; 30%–60% moderate heterogeneity; 50%–90% substantial heterogeneity; 75%–100% considerable heterogeneity (Higgins et al. 2024). A fixed‐effect or random‐effects meta‐analysis model will be selected based on the magnitude of statistical heterogeneity as well as on the clinical and methodological heterogeneity of the included studies. In the presence of low heterogeneity, a fixed‐effect model will be considered appropriate, whereas in cases of moderate to high heterogeneity, a random‐effects model will be applied.

### 2.13 Subgroup or Subset Analyses

Subgroup analyses will be conducted to explore potential sources of heterogeneity, provided there is a sufficient number of studies and data available. The analyses will be conducted considering the type of intervention (therapeutic exercises, manual therapy, electrotherapy, health education), gestational risk (usual risk pregnancy, high‐risk pregnancy), and gestational trimester (first trimester up to 12 weeks, second trimester up to 27 weeks, and third trimester above 28 gestational weeks). In studies that include pregnant women in more than one trimester without stratified analysis, the data will be used considering the average provided by the authors. When this is not possible, the study will not be included in this analysis.

If variability is identified among the therapeutic exercises applied in the studies, other subgroups may be added considering different exercise modalities. Thus, comparisons between subgroups will be performed through the analysis of differences between subgroups, when applicable.

### 2.14 Ethical Considerations

Because of the nature of the study (systematic literature review), ethical approval will not be required.

## 3 Conclusion

LPP is a prevalent condition during pregnancy and represents a clinical challenge due to limitations in the use of medications and the need for interventions that are effective and safe for both the pregnant woman and the foetus. However, the lack of standardisation in clinical trials hinders replication in future studies and limits the incorporation of non‐pharmacological practices into clinical routine.

The use of the TIDieR checklist in this systematic review will allow for the evaluation of both the clinical efficacy and the reporting quality of the proposed interventions, thus promoting greater methodological transparency and scientific reproducibility. This study will allow for discussion not only of which interventions are used, but also of how they are described, broadening the critical understanding of the available literature.

By identifying interventions with a greater level of detail, this review could potentially contribute to translating scientific knowledge into clinical practice in the care of pregnant women with LPP. Thus, healthcare professionals will be able to understand more clearly the fundamental aspects of the interventions, such as frequency, intensity, duration, method of application, and adaptations, favouring safe and effective implementation. A critical analysis of the intervention reports will contribute to the adoption of better structured, individualised, and evidence‐based approaches that respect the specificities of pregnancy, as well as to the development of future research involving well‐described and clinically applicable interventions.

From a social perspective, the results can support the development or improvement of clinical care protocols and care pathways focused on women's health and prenatal care. Promoting qualified care for pregnant women with potential impact on functionality, quality of life, and organisation of health services.

## Author Contributions

**Marília Caroline Ventura Macedo Leite:** conceptualization, methodology, project management, writing – original draft, writing – review and editing. **Mateus Dantas Azevedo de Lima:** conceptualization, methodology, visualization, writing – review and editing. **Rafaela Jéssica Silveira de Souza:** conceptualization, methodology, visualization, writing – review and editing. **Vanessa Patrícia Soares de Souza:** conceptualization, methodology, visualization, writing – review and editing. **Elizabel de Souza Ramalho Viana:** conceptualization, methodology, visualization, writing – review and editing. All authors have read and approved the final version of the manuscript.

## Funding

This work was supported by Coordenação de Aperfeiçoamento de Pessoal de Nível Superior (Grant 88887.145938/2025‐00).

## Conflicts of Interest

The authors declare no conflicts of interest.

## Supporting information


Supporting Information S1

Supporting Information S2

## Data Availability

No datasets were generated or analysed during the current study. Data generated during the systematic review will be available from the corresponding author upon request.
