# Supplementary material for: Non‐Pharmacological Interventions for the Treatment of Lumbopelvic Pain in Pregnant Women: A Systematic Review Protocol Using the TIDieR Checklist
Source: Musculoskeletal Care. 2026 Jul 28;24(3):e70250. doi: 10.1002/msc.70250 (PMC13415757; doi:10.1002/msc.70250)
Supplement: Supplementary file 2 — Supporting Information S2 [file MSC-24-e70250-s001.docx]

**Appendix 2 - Database search strategies**

| **Search strategy** | **Database** |
| --- | --- |
| (("Pregnancy"[Mesh] OR pregnan* OR gestation* OR antenatal OR prenatal) AND ("Low Back Pain"[Mesh] OR "Pelvic Girdle Pain"[Mesh] OR "lumbopelvic pain" OR "low back pain" OR "pelvic girdle pain" OR "pelvic pain" OR "posterior pelvic pain" OR "symphysis pubis dysfunction" OR "pubic symphysis pain" OR "pregnancy-related pelvic girdle pain") AND ("Exercise Therapy"[Mesh] OR "Physical Therapy Modalities"[Mesh] OR "Rehabilitation"[Mesh] OR "Complementary Therapies"[Mesh] OR "motor control" OR stabilization OR stabilisation OR Pilates OR yoga OR strengthening OR "resistance training" OR "therapeutic exercise" OR "exercise therapy" OR "core stability" OR "pelvic stability" OR "neuromuscular training" OR "manual therapy" OR mobilization OR manipulation OR "spinal manipulation" OR education OR "health education" OR electrotherapy OR TENS)) | PubMed |
| pregnancy AND lumbopelvic pain  pregnancy AND pelvic pain  pregnancy AND pelvic girdle pain  pregnancy AND low back pain  pregnancy AND posterior pelvic pain  pregnancy AND symphysis pubis dysfunction | PEDro |
| ([mh Pregnancy] OR pregnan* OR gestation* OR antenatal OR prenatal) AND ([mh "Low Back Pain"] OR [mh "Pelvic Girdle Pain"] OR lumbopelvic pain OR pelvic girdle pain OR pelvic pain OR posterior pelvic pain OR symphysis pubis dysfunction OR pubic symphysis pain OR pregnancy-related pelvic girdle pain) AND ([mh "Exercise Therapy"] OR [mh "Physical Therapy Modalities"] OR [mh "Rehabilitation"] OR [mh "Complementary Therapies"] OR motor control OR stabilization OR stabilisation OR pilates OR yoga OR strengthening OR resistance training OR therapeutic exercise OR exercise therapy OR core stability OR pelvic stability OR neuromuscular training OR manual therapy OR mobilization OR manipulation OR education OR electrotherapy OR TENS) | Biblioteca Cochrane |
| (TITLE-ABS-KEY (pregnancy OR pregnant) AND TITLE-ABS-KEY (pelvic pain OR low back pain OR lumbopelvic pain OR pubic symphysis pain) AND TITLE-ABS-KEY (exercise OR therapy OR intervention OR physiotherapy OR manual therapy) ) | Scopus |
| TS=((pregnan* OR gestation* OR antenatal OR prenatal) AND (lumbopelvic pain OR "low back pain" OR "pelvic girdle pain" OR pelvic pain OR posterior pelvic pain OR "symphysis pubis dysfunction" OR "pubic symphysis pain" OR "pregnancy-related pelvic girdle pain") AND ("motor control" OR stabilization OR stabilisation OR pilates OR yoga OR strengthening OR "resistance training" OR "therapeutic exercise" OR "exercise therapy" OR "core stability" OR "pelvic stability" OR "neuromuscular training" OR "manual therapy" OR mobilization OR manipulation OR education OR electrotherapy OR TENS)) | Web of Science |
| ('pregnancy'/exp OR pregnan*:ti,ab,kw OR gestation*:ti,ab,kw OR antenatal:ti,ab,kw OR prenatal:ti,ab,kw) AND ('low back pain'/exp OR 'pelvic girdle pain'/exp OR lumbopelvic pain:ti,ab,kw OR pelvic pain:ti,ab,kw OR posterior pelvic pain:ti,ab,kw OR 'symphysis pubis dysfunction':ti,ab,kw OR 'pubic symphysis pain':ti,ab,kw OR 'pregnancy-related pelvic girdle pain':ti,ab,kw) AND ( 'exercise therapy'/exp OR 'physical therapy'/exp OR 'rehabilitation'/exp OR 'complementary medicine'/exp OR 'manual therapy'/exp OR 'joint mobilization'/exp OR 'spinal manipulation'/exp OR 'acupuncture therapy'/exp OR electrotherapy/exp OR 'health education'/exp OR motor control:ti,ab,kw OR stabilization:ti,ab,kw OR stabilisation:ti,ab,kw OR pilates:ti,ab,kw OR yoga:ti,ab,kw OR strengthening:ti,ab,kw OR 'resistance training':ti,ab,kw OR 'therapeutic exercise':ti,ab,kw OR 'exercise therapy':ti,ab,kw OR 'core stability':ti,ab,kw OR 'pelvic stability':ti,ab,kw OR 'neuromuscular training':ti,ab,kw OR manual therapy:ti,ab,kw OR mobilization:ti,ab,kw OR manipulation:ti,ab,kw OR education:ti,ab,kw OR electrotherapy:ti,ab,kw OR TENS:ti,ab,kw) | Embase |
| (tw:(pregnan* OR gestante* OR gravidez OR antenatal OR prenatal)) AND (tw:("dor lombopélvica" OR lombalgia OR "low back pain" OR "pelvic girdle pain" OR "dor pélvica" OR "disfunção da sínfise púbica" OR "pubic symphysis pain" OR "pregnancy-related pelvic girdle pain")) AND (tw:("controle motor" OR estabilização OR Pilates OR ioga OR fortalecimento OR "treinamento de resistência" OR "terapia com exercícios" OR "exercício terapêutico" OR "estabilidade do core" OR "estabilização pélvica" OR "treinamento neuromuscular" OR "terapia manual" OR mobilização OR manipulação OR educação OR eletroterapia OR TENS)) | LILACS |
